# Prevalence of chronic kidney disease and risk factors in North-Central Nigeria: a population-based survey

**DOI:** 10.1186/s12882-020-02126-8

**Published:** 2020-11-10

**Authors:** Timothy Olusegun Olanrewaju, Ademola Aderibigbe, Ademola Alabi Popoola, Kolawole Thomas Braimoh, Mikhail Olayinka Buhari, Olanrewaju Timothy Adedoyin, Sulyman Alege Kuranga, Sikiru Abayomi Biliaminu, Adindu Chijioke, Abdulwahab Akanbi Ajape, Diederick E Grobbee, Peter J Blankestijn, Kerstin Klipstein-Grobusch, the Ilorin Renal Study Group

**Affiliations:** 1 Division of Nephrology, Department of Medicine, University of Ilorin and University of Ilorin Teaching Hospital, Ilorin, Nigeria; 2 Julius Global Health, Julius Center for Health Sciences and Primary Care, University Medical Center Utrecht, Utrecht University, Utrecht, The Netherlands; 3 Division of Nephrology, Department of Medicine, University of Ilorin Teaching Hospital, Ilorin, Nigeria; 4 Division of Urology, Department of Surgery, University of Ilorin and University of Ilorin Teaching Hospital, Ilorin, Nigeria; 5 Department of Radiology, University of Ilorin and University of Ilorin Teaching Hospital, Ilorin, Nigeria; 6 Department of Pathology, University of Ilorin and University of Ilorin Teaching Hospital, Ilorin, Nigeria; 7 Department of Paediatrics, University of Ilorin and University of Ilorin Teaching Hospital, Ilorin, Nigeria; 8 Department of Chemical Pathology, University of Ilorin and University of Ilorin Teaching Hospital, Ilorin, Nigeria; 9 Department of Nephrology and Hypertension, University Medical Center Utrecht, Utrecht University, Utrecht, The Netherlands

**Keywords:** Chronic kidney disease, Risk factors, Hypertension, Diabetes, Obesity, Sub-Saharan Africa, Nigeria

## Abstract

**Background:**

Chronic kidney disease (CKD) is a growing challenge in low- and middle-income countries, particularly in sub-Saharan Africa. There is insufficient population-based data on CKD in Nigeria that is required to estimate its true burden, and to design prevention and management strategies. The study aims to determine the prevalence of CKD and its risk factors in Nigeria.

**Methods:**

We studied 8 urban communities in Kwara State, North-Central zone of Nigeria. Blood pressure, fasting blood sugar, urinalysis, weight, height, waist circumference and hip circumference were obtained. Albuminuria and kidney length were measured by ultrasound while estimated glomerular filtration rate (eGFR) was derived from serum creatinine, using chronic disease epidemiology collaboration (CKD-EPI) equation. Associations of risk factors with CKD were determined by multivariate logistic regression and expressed as adjusted odds ratio (aOR) with corresponding 95% confidence intervals.

**Results:**

One thousand three hundred and fifty-three adults ≥18 years (44% males) with mean age of 44.3 ± 14.4 years, were screened. Mean kidney lengths were: right, 93.5 ± 7.0 cm and left, 93.4 ± 7.5 cm. The age-adjusted prevalence of hypertension was 24%; diabetes 4%; obesity 8.7%; albuminuria of > 30 mg/L 7%; and dipstick proteinuria 13%. The age-adjusted prevalence of CKD by estimated GFR < 60 ml/min/1.73m^2^ and/or Proteinuria was 12%. Diabetes (aOR 6.41, 95%CI = 3.50–11.73, *P* = 0.001), obesity (aOR 1.50, 95%CI = 1.10–2.05, *P* = 0.011), proteinuria (aOR 2.07, 95%CI = 1.05–4.08, *P* = 0.035); female sex (aOR 1.67, 95%CI = 1.47–1.89, *P* = 0.001); and age (aOR 1.89, 95%CI = 1.13–3.17, *P* = 0.015) were the identified predictors of CKD.

**Conclusions:**

CKD and its risk factors are prevalent among middle-aged urban populations in North-Central Nigeria. It is common among women, fueled by diabetes, ageing, obesity, and albuminuria. These data add to existing regional studies of burden of CKD that may serve as template for a national prevention framework for CKD in Nigeria. One of the limitations of the study is that the participants were voluntary community dwellers and as such not representative for the community. The sample may thus have been subjected to selection bias possibly resulting in overestimation of CKD risk factors.

## Background

Chronic kidney disease (CKD) is a significant health concern globally. The Global Burden of Disease (GBD) study ranked CKD the 19th leading cause of morbidity and death in 2013 [1]. Worldwide, the age-standardized CKD prevalence is 10.4% in men and 10% in women and is higher in low- and middle-income countries than high-income countries [2]. For sub-Saharan Africa (SSA), recent systematic reviews reported a prevalence of 13.9% [3], and 10.1% respectively [4]; The pooled prevalence for CKD was 16% in West Africa, the highest in the continent. CKD is characterized by young age of patients in Africa, huge morbidity and premature deaths. About 90% of patients with CKD die within 90 days of starting dialysis.

Despite the large population size of Nigeria with 180million people, little is known about the epidemiology of CKD in the general population. There is no national data on prevalence of CKD, and only few community-based studies were done in some regions of the country. A recent systematic review identified 7 population-based studies, 5 from the Southern part and 2 from the Northern part [5]; in this study, the prevalence of CKD ranged from 2.5 to 26%. Oluyombo et al. [6] previously reported the prevalence of CKD of 18% in a rural community in South-Western Nigeria. Similar study in the South-East Nigeria found a prevalence of 11.4% in rural, and 11.7% in semi-urban dwellers [7]. In addition, a study from North-West Nigeria in the recent review documented CKD prevalence of 26% [5], suggesting overall high prevalence of CKD and indicating a need for more studies to understand the true burden of CKD in Nigerian population. Combining regional population-based studies with sufficient power allow to estimate the true magnitude of CKD where national data is lacking. Such aggregate data permit adjustment for confounding factors such as ethnicity, and prevalent risk factors in the regions. Nigeria is a multi-ethnicity nation with diverse socio-cultural practices and economic status that affect disease pattern, thus findings of few CKD studies in different regions are inconsistent. Notably, studies from North-Central comprising more than 25million people are scarce. We undertook this study to generate a crude estimate of CKD in a fairly large population of urban dwellers in a state within the North-central region of Nigeria. Kwara state is a home to the major ethnic groups in Nigeria and therefore the findings may inform the magnitude and pattern of occurrence of CKD in the country.

The aim of the current study was to estimate the prevalence of CKD and cardiovascular disease risk factors, and determine risk factors for CKD, among the adult population in Kwara State located within the North-Central geo-political zone of Nigeria.

## Methods

### Study design and setting

We conducted a cross-sectional study in eight urban communities in Kwara State located in North-Central zone of Nigeria. The study was conducted by the Ilorin Renal Study Group (IRSG) with the supports of the University of Ilorin Teaching Hospital, the local government authority and staff of the primary care hospital of the host community. The IRSG comprises adult and Paediatric Nephrologists, Nephrologists-in-training, Nephrology Nurses, Laboratory Scientists and Technicians, Radiologists, Urologists and Pathologists with interest in CKD care. They were responsible for administering questionnaires, physical measurements, and blood and urine collection for laboratory tests. The staff of the hospitals and health centers of the host communities were involved in the registration, physical measurements and data collection from the participants. The study was conducted in 8 urban communities. The study was undertaken as a community screening programme during world kidney days. The 8 communities were randomly selected by lottery within the 16 local governments that made up the Kwara State.

The communities were capitals of local government areas (LGA) randomly selected from 16 that constitute Kwara State and they are: Adewole (Ilorin Central), Omu-Aran (Irepodun LGA), Oke-Oyi (Ilorin East LGA) Shao (Shao LGA) Offa (Offa LGA), Pake (Ilorin Central LGA), Jebba (Jebba LGA), and Afon (Oyun LGA). The people are homogenous with predominant ethnic groups comprising Yoruba, Fulani and Igbo. Other tribes were Nupe and Baruba. Majority are civil servants, traders, artisan and students. The population of Kwara state is about 3 million. The programme was announced on the radio and through the community leaders. We obtained approval from the local government authority and the traditional heads of each community 12 weeks before the screening.

### Study population

The study population were healthy adults 18 years and older who are resident in the host community. The participants were voluntary individuals mobilized through the community heads and local government’s heads. Health education on kidney disease was delivered and Verbal informed consent was obtained from each participants.

We excluded people with urinary tract infection and women that are menstruating which may produce false positive albuminuria and hematuria. We also excluded pregnant women which may yield physiological albuminuria.

### Measurements of variables

All participants filled in a questionnaire about demographic characteristics and medical history, and were physically examined. We measured blood pressure by mercury sphygmomanometer with standard cuff (25cmx12cm), on the right arm with participants in sitting position after 5 min rest. Two measurements were taken and the average was recoded. The weight was measured with a Seca weighing scale placed on a flat, hard surface with the participants wearing light clothing. The height was measured by a stadiometer with the participants standing without shoes. We calculated body mass index (BMI) by weight (kg)/height (m^2^). The waist circumference (WC) was measured with a non-elastic graduated tape at midpoint between the lower margin of the lowest palpable rib and the top of the iliac crest. Hip circumference (HC) was measured at the widest portion of the buttocks. We calculated waist-hip ratio (WHR) by WC (cm) / HC (cm). One sonologist performed ultrasonography of the kidney using Sonoscape Medical Digital Colour Doppler Ultrasound System (S30) with multiple frequency probes. Albuminuria was determined by HemoCue Albumin 201 point-of-care system (HemoCue America, CA) [8]. Fasting blood was taken and analyzed for sugar, creatinine and haemoglobin were measured at pathology laboratory of University of Ilorin Teaching Hospital. Blood samples for creatinine were collected in heparinized bottles and analyzed by Jaffe’s reaction method using RA-50 spectrophotometer (Bayer, Germany) at Chemical Pathology Laboratory of the hospital. Fasting plasma glucose samples were collected in a fluoride oxalate bottle and analyzed by glucose oxidase method at the same laboratory. Also, blood samples for haemoglobin were collected in heparinized bottles and measured by Sysmex XP-300™ automated haematology analyzer (Kope, Japan), at hematology laboratory. We performed dipstick test onsite for proteinuria and haematuria. Participants were given sterilized plain bottles to collect 10 mL of early morning, clean catch urine. The urine was examined physically and tested with reagent strips (Multistix®10 SG; Bayer, Leverkusen, Germany). Haematuria and proteinuria were read within the time indicated by the manufacturer*.*

### Variables of interest and definitions

Estimated glomerular filtration rate (eGFR) was derived from chronic disease epidemiology collaboration (CKD-EPI) [9]. Serum creatinine was estimated by Jaffe’s method. We defined CKD stages by kidney disease outcomes quality initiative classification, eGFR < 60 ml/min/1.73m^2^ and/or albuminuria > 300 mg/g, hypertension as blood pressure ≥ 140/90 mmHg or use of antihypertensive drugs, diabetes as fasting plasma glucose > 7.0 mmol/L, and obesity as BMI ≥30 kg/m^2^ or waist-hip ratio > 0.85 in women and > 1.0 in men. Dipstick proteinuria was reported as ≥ + 1, and hematuria as ‘trace’ or ≥ + 1. Albuminuria was defined as > 30 mg/g, and anaemia by hemoglobin levels < 12 g/dl for women, and < 13 g/dl men.

### Data analysis

The data were analyzed by SPSS version 17 (SPSS Inc.; Chicago, IL, USA). The missing data was handled by multiple imputation method using Markov chain Monte Carlo (MCMC) method.

Continuous variables that were normally distributed are expressed in means with standard deviation (SD), while those that were skewed are expressed in median with range. Categorical variables are expressed as percentages. Student t test was used to determine differences in the means of normally-distributed continuous variables and Chi-squared test for categorical variables. The prevalence of CKD and risk factors were adjusted for age and sex by direct standardization techniques, using the age and sex structure (stratification) of the 2006 population census of Nigeria [10]. Partial Spearman correlation coefficient was used to determine independent relationship between the variables and decreased eGFR. Factors that drive CKD in Nigeria is yet to be fully understood. Among the measured characteristics of the participants, some may be associated with CKD, thus the application of correlation analysis. Those that correlated were entered into multivariate logistic regression model to identify independent determinants of CKD. We determined the predictors of CKD by multivariate logistic regression using a stepwise forwards method. Results were expressed as odd ratio (OR) and adjusted odds ratio (aOR) with corresponding 95% confidence interval (CI). A two-sided *p*-value < 0.05 was chosen as significant level.

## Results

### Baseline characteristics of the study population

Table 1 shows the baseline demographic and clinical characteristics of the study population by gender. The mean age was 44 ± 14 years and higher in men than women (*p* = 0.007). Women accounted for 56% (*n* = 758) of the participants. The prevalence of self-reported hypertension was 8.6%, and was higher in women (10%) than men (5.9%), *p* = 0.002. We observed a prevalence of hypertension of 20.6% with no difference between men and women (*p* = 0.457). Similarly, no difference in the prevalence of self-reported (1%) and observed (6%) diabetes was observed between men and women. Women presented with higher body mass index (BMI, 27.2 ± 6.4 versus 24.5 ± 4.4 kg/m^2^, *p* = 0.001), and waist circumference (92.07 ± 18 versus 86.18 ± 18 cm, *p* = 0.001) than men, while men had higher proteinuria (15.3% versus 11.1%, *p* = 0.002), haemoglobin (13.1 ± 1.2 versus 12.6 ± 1.1 g/dL, *p* = 0.001), and eGFR (106(75–156) versus 85(61–114) ml/min/1.73 m2, *p* = 0.031).

**Table 1:** Characteristics of the study participants (*n* = 1353)

Characteristics	Total (*n* = 1353)	Males (*n* = 595)	Females (*n* = 758)	***p*** value
Age (years)	44.3 ± 14.4	45.5 ± 14.2	43.4 ± 14.4	0.007
Hypertension (reported), n(%)^a^	116 (8.6)	35 (5.9)	81 (10.7)	0.002
Hypertension measured, n(%)^a^	279 (20.6)	126 (21.2)	153 (20.2)	0.457
Diabetes, (reported), n(%)^a^	14 (1.0)	7 (1.2)	7 (0.9)	0.648
Diabetes measured, n(%)^a^	82 (6.1)	30 (5.4)	52 (6.6)	0.351
Fasting blood sugar (mmol/L)	5.8 (2.0)	5.8 (1.8)	5.8 (2.2)	0.784
Systolic Blood pressure (mmHg)	129 (22)	129 (21)	128 (22)	0.306
Diastolic Blood Pressure (mmHg)	80 (13)	81 (13)	79 (13)	0.161
Weight (Kg)	70.3 (15.9)	70.1 (16.7)	69.7 (18.5)	0.092
Height (m)	1.63 (0.1)	1.69 (0.2)	1.59 (0.2)	0.001
Body Mass Index (Kg/M^2^)	26.0 (5.8)	24.5 (4.4)	27.2 (6.4)	0.001
Waist Circumference (cm)	89.12 (18)	86.18 (18)	92.07 (18)	0.001
Hip circumference (cm)	91.69 (18)	89.26 (18)	94.12 (18)	0.407
Waist-hip ratio	0.97 (0.3)	0.96 (0.4)	0.98 (0.2)	0.845
Proteinuria (dipstick), n(%)^a^	175 (12.9)	91 (15.3)	84 (11.1)	0.002
Albuminuria (mg/L)^b^	30 (16–46)	28 (16–44)	30 (16–47)	0.805
Haemoglobin (g/dL)	12.9 (1.3)	13.3 (1.2)	12.6 (1.1)	0.001
Left kidney (mm)	93.4 (7.5)	93.7 (10.7)	92.0 (7.9)	0.030
Right Kidney (mm)	93.5 (7.0)	94.4 (8.7)	92.8 (7.3)	0.002
Creatinine (umol/L)^b^	80 (63–106)	84 (65–109)	77 (59–103)	0.107
eGFRCKD-EPI (mL/min/1.73m^2^)^b^	96.3 (69–126)	106 (75–106)	85 (61.114)	0.031

*eGFR* estimated glomerular filtration rate, *CKD-EPI* chronic kidney disease-Epidemiology collaboration, *IQR* Inter quarter range

^a^Chi square test

^b^Median (IQR)

### Prevalence of CKD and risk factors

Table 2 shows the age and sex-adjusted prevalence of CKD and risk factors in the study population. The overall prevalence of CKD in the community was 12% and significantly higher in women (14.1%) than men (9.5%), *p* = 0.001. Stage 3 CKD accounted for the highest prevalence (9.5%) and was significantly higher in women (11.6%) than men (6.9%), *p* = 0.001.Prevalence was observed to increase with age from 10.5% at age < 30 years to 40-49 years (14.7%), and declined slightly to 11.7% at > 50 years, showing a similar trend in men and women. Albuminuria of ≥30 mg/dL was found in 7.1%, and was similar between men and women, *p* = 0.090. The prevalence of hypertension was 23.7%, diabetes 4.1% and are similar in men and women. Obesity was observed in 8.7% of the study population and was significantly higher in women (11.8%) than men (5.2%), *p* = 0.001.

**Table 2:** Age- and sex-adjusted prevalence of CKD, and risk factors in the study population (*n* = 1353, Males = 595; Females =758)

Characteristic	Total(%)	Male (%)	Female (%)	X^**2**^	***P*** value
**CKD eGFR < 60 ml/min/1.73m**^**2**^**,**	11.8	9.5	14.1	16.6	< 0.001
Stage 3 (estimated GFR 30 to 59)	9.5	6.9	11.6	14.79	< 0.001
Stage 4 (estimated GFR 15–29)	1.3	1.29	1.4	0.79	0.373
Stage 5 (estimated GFR < 15)	1.0	1.1	0.9	0.59	0.367
**Age**					
< 30	10.5	9.6	10.9	4.89	0.027
30–39	8.8	6.98	10.9	5.92	0.015
40–49	14.7	11.4	17.6	5.73	0.017
> 50	11.7	19.5	14	4.17	0.041
**Albuminuria**					
≥ 30 mg/dL	7.1	7.3	6.9	0.28	0.598
**Hypertension**					
SBP ≥140 mmHg+/or DBP ≥90 mmHg	23.7	25.2	23.4	0.306	0.442
**Diabetes**					
FBS ≥7.0 mmol/L	4.1	3.8	4.5	0.869	0.351
**Overweight and obesity**					
25 to 29.9 kg/m^2^, n (%)	15.3	15	16	0.804	0.459
≥ 30 kg/m^2^, n (%)	8.7	5.2	11.8	39.72	< 0.001

*CKD* chronic kidney disease, *eGFR* estimated glomerular filtration rate

### Participants with CKD compared with those without CKD

Characteristics of participants with CKD compared with those without CKD are shown in Table 3. Participants with CKD were older (46 ± 12 versus 42 ± 14 years, *p* = 0.042), had more often diabetes (crude prevalence 9.1% versus 5.4%, *p* = 0.043), wider hip circumference (98.29 ± 16.94 versus 92.27 ± 17.99 cm, *p* = 0.01), higher median albuminuria (38.6(15.2–59.4) versus 30.9(13.2–55) mg/L, *p* = 0.042), and lower eGFR (44.8(32.3–54.9) versus 104.5(85.2–132.5) ml/min/1.73m^2^, *p* = 0.001. The figures were comparable between men and women. There was no difference in mean systolic blood pressure (*p* = 0.564), diastolic blood pressure (*p* = 0.543), prevalence of hypertension (*p* = 0.512), obesity (*p* = 0.266), and dipstick proteinuria (*p* = 0.591).

**Table 3:** Characteristics of participants with and without CKD (*n* = 1353)

Characteristic	CKD (***n*** = 236)	no-CKD (***n*** = 1117)	***p*** value
Gender (Male), n(%)	80 (33.9)	515 (46.1)	0.001
Age (years)	46 (12)	42 (14)	0.045
Hypertension (reported), n(%)	25 (10.8)	91 (8.1)	0.188^a^
Hypertension measured, n(%)	52 (22)	227 (20.3)	0.512^a^
Systolic Blood pressure (mmHg)	129 (21)	0.564	128 (22)
Diastolic Blood Pressure (mmHg)	81 (11)	80 (13)	0.543
Diabetes, (reported), n(%)	3 (1.3)	11 (0.9)	0.566^a^
Diabetes measured, n(%)	21 (9.1)	61 (5.4)	0.048^a^
Fasting blood sugar (mmol/L)	6.17 (2.5)	6.02 (2.2)	0.603
Weight (Kg)	70.5 (15)	70.3 (16)	0.868
Body Mass Index (Kg/M^2^)	26.5 (5.4)	26.2 (5.7)	0.607
Obesity (BMI ≥ 30Kg/M^2^)	35 (15.1)	139 (12.4)	0.266^a^
Waist Circumference (cm)	91.76 (17.8)	91.43 (18.8)	0.907
Hip circumference (cm)	98.3 (16.9)	92.3 (17.9)	0.01
Waist-hip ratio	0.95 (0.22)	1.00 (0.32)	0.208
Proteinuria (dipstick), n(%)	27 (11.4)	148 (13.2)	0.591^a^
Albuminuria (mg/L)^b^	38.6 (15.2–59.4)	30.9 (13.2–55.0)	0.042
Haemoglobin (g/dL)	12.8 (1.3)	12.9 (1.3)	0.706
Left kidney (mm)	93.3 (7.4)	93.4 (7.5)	0.911
Right Kidney (mm)	93.5 (7.6)	93.4 (7.7)	0.988
Creatinine (umol/L)^b^	146 (122–178)	80 (59–106)	0.001
eGFRm-CKD-EPI (mL/min/1.73m^2^)^b^	45.9 (34.4–55.3)	107.8 (87.8–135.9)	0.001
eGFRf-CKD-EPI (mL/min/1.73m^2^)^b^	43.9 (31.9–54.9)	102.9 (83.2–129.1)	0.001
eGFR-CKD-EPI (mL/min/1.73m^2^)^b^	44.8 (32.3–54.9)	104.5 (85.2–132.5)	0.001

*CKD* chronic kidney disease, *eGFR* estimated glomerular filtration rate, *CKD-EPI* chronic kidney disease-Epidemiology collaboration, *eGFRm* eGFRm for males, *eGFRf* eGFRm for females

^a^Chi square test

^b^Median (IQR)

### Correlation of clinico-demographic factors with eGFR for participants with CKD

The correlation between demographic and clinical factors with eGFR is described in Table 4. Variables that correlated significantly were age (*r* = − 0.171, *p* = 0.001), female sex (*r* = 215, *p* = 0.001), measured hypertension (*r* = − 0.137, *p* = 0.001), diabetes (*r* = − 0.216, *p* = 0.001), dipstick proteinuria (*r* = − 0.074, *p* = 0.010), albuminuria (*r* = − 0.057, *p* = 0.038), waist circumference (*r* = − 0.073, *p* = 0.008), hip circumference (*r* = − 0.230, *p* = 0.001), and waist-hip ratio (*r* = − 0.198, *p* = 0.001).

**Table 4:** Correlation of variables with eGFR < 60 ml/min/1.73m^2^ in the participants

Characteristic	Correlation coefficients (r)	***P*** value
Age	−0.171	< 0.001
Sex	0.215	< 0.001
Hypertension (reported)	−0.021	0. 451
Hypertension (measured)	−0.137	< 0.001
Systolic blood pressure	−0.117	0.005
Diastolic blood pressure	−0.094	0.022
DM reported	−0.025	0.367
DM measured	−0.216	< 0.001
Fasting blood sugar	−0.032	0.237
Urine proteinuria	−0.074	0.010
Albuminuria	0.057	0.038
Urine blood	−0.006	0.826
Body mass index	−0.093	0.065
Waist circumference	−0.073	0.008
Hip circumference	−0.230	< 0.001
Waist hip ratio	−0.198	< 0.001
Haemoglobin	0.027	0.641
Right kidney	0.033	0.227
Left kidney	0.015	0.581

*eGFR* estimated glomerular filtration rate

### Risk factors for CKD

Independent risk factors for CKD determined by multiple logistic regressions are shown in Table 5. Diabetes (aOR = 6.41, 95CI = 3.50–11. 73, *p* = 0.001) was the most potent risk factor for CKD in the community followed by age (aOR = 1.89, 95%CI = 1.13–3.17, *p* = 0.015), female sex, and central obesity defined by waist-hip ratio. Other traditional risk factors for CKD such as hypertension and obesity did not contribute to the logistic regression model. Among the markers of kidney damage assessed in the study, dipstick proteinuria (aOR = 2.07, 95%CI = 1.05–4.08, *p* = 0.035) and quantitative albuminuria (aOR = 1.47, 95%CI = 1.10–1.96, *p* = 0.009) were significantly associated with CKD.

**Table 5:** Multivariate logistic regression^a^ of risk factors for CKD^b^

Characteristics	Crude OR^c^ (95%CI)	Adjusted OR (95%CI)	Pvalue
Age	1.31 (1.06–1.62)	1.89 (1.13–3.17)	0.015
Sex (Female)	1.76 (1.29–2.40)	1.67 (1.47–1.89)	0.001
Urine Protein	2.06 (1.04–4.07)	2.07 (1.05–4.08)	0.035
Albuminuria	1.74 (1.13–3.24)	1.47 (1.10–1.96)	0.009
Obesity (waist hip ratio)	2.19 (1.62–2.98)	1.49 (1.09–2.05)	0.011
Diabetes	5.02 (3.24–7.77)	6.41 (3.49–11.73)	0.001

^a^The multiple logistic regression model adjusted for variables that correlated significantly with eGFR < 60 ml/min/1.73m^2^ in Table 4 (measured hypertension, systolic high blood pressure, waist circumference, hip circumference, and waist hip ratio)

^b^*CKD* chronic kidney disease

^c^*OR* odds ratio

## Discussions

### Prevalence of CKD

This study estimated the prevalence of chronic kidney disease and its risk factors in urban populations of Kwara State in Nigeria. CKD, its risk factors and markers of kidney damage were prevalent among adults in Kwara State and occur in the younger population. CKD affected more women than men; age, female sex, diabetes, and obesity were the most pronounced risk factors for CKD.

The prevalence of CKD of 12% in this study is similar to the adjusted figures reported by Ulasi et al. (11.4%) [7], and significantly higher than report by Okwuonu et al. (7.6%) [11]. Both studies were conducted in semi-urban communities in South-East zone of Nigeria while our study was in urban communities. Ulasi et al. [7] adjusted the prevalence for age and sex, making it more comparable to our findings, and they studied 1941 participants. The reduced sample size of 328 participants may account for the lower prevalence reported by Okwuonu et al. [11]. The prevalence in our study is also comparable to the unadjusted prevalence of CKD reported by Oluyombo et al. (12.3%) [6] in South-West Nigeria. Overall, there seems to be no significant difference in the prevalence of CKD between similar large sample-sized studies in Southern and North-Central zones of Nigeria.

Prevalence of CKD is further comparable to global and African figures [2, 4, 12] in general. CKD prevalence in our study compares well with CKD prevalence in Kinshasa (12.4%), an urban population in democratic republic of Congo [13], urban city of Cameroon (11%) [14], but is considerably higher than reported in Uganda (6%) [15]. Conversely, Stanifer et al. [16] reported 15.2% in urban population of Tanzania and a recent study in Senegal showed CKD prevalence to be three-fold higher than in our study [17]. Overall, the prevalence of CKD is high [3, 4] and varies among Sub-Saharan African populations.

The discrepancies in the prevalence may be due to different equations used to derive estimated glomerular filtration rate (eGFR), study design and population. Some studies used Modification of Diet in Renal Disease (MDRD) or Chronic Kidney Disease Epidemiology Collaboration (CKD-EPI), others used Cochroft-Gault equation which consistently overestimates CKD in the general population. Further, many studies employed a cross sectional design with a single measure of eGFR.

The high prevalence of CKD in SSA is due to many factors including a growing trend of non-communicable disease, prevalent use of nephrotoxic herbs and drugs, human immunodeficiency virus infection, and obesity. In addition, ethnicity and genetic factors such as inheritance of risk alleles of APOL1 may also contribute to the CKD risk. As observed in many previous studies in SSA, patients with CKD in this study are much younger than those in high-income countries, and this has implications for economic productivity of the sub-region.

### CKD and gender distribution

The higher prevalence of CKD we observed in women agrees with other studies in Nigeria and global analysis [2, 5, 18–20]. Large population-based studies have shown that prevalence of CKD is greater in women than in men, regardless of age; for example, data from National Health and Nutrition Examination Survey (NHANES) showed that CKD is more frequent among women in United State population [20]. Some of the reasons why women could be predispositioned to higher CKD risk include, pregnancy associated kidney damage, higher obesity, subclinical autoimmune diseases, and use of nephrotoxic cosmetic agents [20]. A multi-county population study including Nigeria reported the prevalence of pregnancy hypertension (gestational hypertension and pre-ecclampsia) of approximately 10%, which is comparable with India, Pakistan and Mozambique [21]. The women were young with median age of 27(23-31 years. One-third (3.4%) presented at antenatal, 3.7% postpartum, and 3.0% had pre-ecclampsia with ecclampsia.

Herbal preparations and mercury containing cosmetic products which are nephrotoxic, are commonly used among women in Nigeria and Africa, and may add to the increased prevalence of CKD among women. Li et al. [22] reported that 81.6% of 748 women in Ibadan, Nigeria used traditional herbal products. They use them mainly for fever, pile, malaria, pregnancy, stomach ache and for prevention of disease. Similarly, Okoronkwo et al. [23] documented that 84.7% of their 732 respondents had used herbal-based alternative medicine and majority were women. In addition, Ulasi et al. [7] had shown that traditional medicine is a risk factor for CKD while mercury-containing soaps also correlated with CKD.

### Prevalence of risk factors for CKD

#### Hypertension

The high prevalence of hypertension corroborates previous studies in Nigeria [24, 25]. Hypertension is a growing public health issue in Nigeria. The high age- and sex-adjusted prevalence of hypertension in this study is consistent with global reports [26]. In this study, self-reported hypertension is low compared with the figure we measured, indicating low awareness of the disease. Low awareness and poor control of hypertension are common, and may be driving hypertension-related complications including CKD in low- and middle-income countries [27, 28].

#### Diabetes

In this study, the prevalence of diabetes is four-fold higher than self–reported, and has highest odds for CKD risk. We also found awareness rate for diabetes of 25%, perhaps underlining high CKD burden. A systematic review and meta-analytic studies revealed that in Nigeria, age-adjusted prevalence of diabetes increased from 2% in 1990 to 5.7% in 2015 with women having higher prevalence compared with men [29]. Diabetes was main predictor of CKD in the earlier study in South-West Nigeria [6] as compared to hypertension reported in the South-East [7]; and it is the commonest cause of CKD worldwide, particularly in high income countries. Globally, diabetes is projected to increase by 50% between 2011 and 2030 with major increments in low- and middle income countries [30]. Nigeria, the most populous country in the continent may record the highest number of affected persons. Thus, the prevalence of CKD may increase in parallel. Therefore, health authorities need to implement prevention programs such as community awareness and screening of the general population to reduce impacts of diabetes.

#### Obesity

We found prevalence of obesity of 8.7%; 5.2% in men, and 11.8% in women. A population study also showed that the obesity is higher in women (36.0%; 95% CI, 32.8–39.4%) than men (19.6%; 95% CI, 17.3–22.2%). In addition, the study of obesity reported that female sex was associated with higher risk for obesity (OR 2.21; 95% CI, 1.73–2.83) [31]. The prevalence of obesity in this study is less than 14.9 and 14.6% previously reported in South-East and South-West Nigeria respectively [6, 7]. This difference in prevalence between the northern and south Nigerians has been consistently observed and may be due to differences in socioeconomic status and lifestyles of the regions [32]. Several studies have identified obesity as a risk factor for CKD [33]. Obesity is a public health problem worldwide affecting adults and teenagers. It is a finding that requires effective lifestyle changes to prevent kidney effects.

#### Markers of kidney damage

We found dipstick proteinuria in 12.9% of the study population, to be higher in men (15.3%) than women (11.1%), despite a higher CKD prevalence in women. No significant difference between participants with CKD and without CKD was observed. Possible explanations include coexisting factors promoting progression of CKD in women that are not measured in this study. For example, use of bleaching soaps and cream that contains mercury is common among women in Nigeria and SSA. We also observed albuminuria ≥30 mg in 7.1% of the study population, with similar proportion among the gender. Studies have consistently shown that albuminuria or proteinuria predicts end-stage kidney disease (ESKD), death and progression of CKD [34, 35]. Therefore, use of antiproteinuric drugs like angiotensin converting enzyme inhibitors is necessary to reduce CKD progression to ESKD.

#### Predictors of CKD in the study population

Determinants of CKD identified in this study were: diabetes, increasing age, and abdominal obesity which are also risk factors for cardiovascular disease (CVD), in line with results from studies elsewhere [11, 36, 37]. Risk factors often coexist in both the general population and high–risk individuals. We previously reported coexistence of at least two of traditional CVD risk factors in the Nigerian population [38] and a recent study showed significant clustering of these risk factors with albuminuria and when eGFR is < 45 ml/min/1.73m^2^ in semi-urban communities in South-west Nigeria [39]. The premature deaths associated with CKD in Nigeria and Africa may be fueled by clustering of CVD risk factors. Apart from age, these risk factors are modifiable, thus providing opportunity to apply prevention and control strategies. Although diabetes was observed to be associated with CKD, glomerulonephritis and hypertension are more commonly reported causes of CKD in Nigeria and Africa. Recently, CKD of unknown etiology is emerging as a significant fraction of the overall CKD pool. CKD due to Specific glomerulonephritis is difficult to characterize in Africa because of low rates of kidney biopsy especially in sub-Saharan Africa. Nevertheless, a review of histologically-proven glomerulonephritis in Africa showed that the primary histology were minimal change disease (16.5% (95%CI: 11.2–22.6)), focal segmental glomerulosclerosis (15.9% (11.3–21.1)), and mesangiocapillary (11.8% (9.2–14.6)) [40]. Furthermore, the use of herbal remedies, nephrotoxic agents and drugs such as non-steroidal anti-inflammatory drugs are prevalent in Africa, and may account for the undetected cause of CKD in the sub-continent. This suggests that the finding of association of diabetes with CKD despite its low incidence in this study, diabetes is not the predominant cause of CKD in Nigeria.

The finding that hypertension is not associated with CKD in this study is surprising because it is well established risk factor for CKD. However, our finding agree with report by Ulasi et al. [7] who also did not find hypertension or diabetes as a predictor of CKD in their 1941 population-based study in South-East Nigeria. Okwuonu et al. [11] however, reported hypertension and not diabetes as a predictor of CKD in same South-East zone with a 328 participants of similar semi-urban dwellers. Furthermore, Oluyombo et al. [6] found a strong association of diabetes with CKD (similar to our study), but a very weak association of hypertension with CKD (OR, 1.038 (CI, 1.006–1.071)) in a 454 rural community in South-West zone of Nigeria. Thus, there is a conflicting report of association of hypertension with CKD in Nigeria which requires further study. The design of our study which is a community survey may account for the observation of non-association of hypertension with CKD.

### Limitations and strengths of the study

Our study provides estimated age- and sex-adjusted prevalence of CKD and its risk factors in adult urban populations in Kwara State, located within the North-Central zone of Nigeria. The strength of the study is the large sample size from multiple communities in previously understudied population. To the best of our knowledge, this study is the largest of the few population-based studies on CKD in Nigeria. Due to the relatively non-diversity in the socio-cultural and life-style of the people in the North-Central and the North generally, the findings may reflect CKD prevalence and risk factors within the region. Thus, it contributes significant proportion to the available data that may be used for health planning.

The limitation of the study is that, we used a cross-sectional (survey) design, and a single creatinine measurement to estimate eGFR which may overestimate the prevalence of CKD. Major guidelines have recommended measuring serum creatinine twice, 3 months apart to validate its level for eGFR estimation. Several studies including analysis of population-based data observed that majority of surveys did not repeat eGFR estimation after 3 months to confirm CKD. However, we included albuminuria with reduced eGFR to define CKD to reduce overestimation of CKD using only eGFR. The participants in this study were voluntary community dwellers and as such not representative for the community and the sample may have been subjected to selection bias possibly resulting in overestimation of CKD risk factors.

## Conclusions

This multi-community study reveals that CKD and its risk factors are prevalent among middle age healthy urban population in North-Central Nigeria. CKD is common among women, fueled by diabetes, aging, obesity, and albuminuria. These data add to existing regional studies on burden of CKD, and may provide template for planning national prevention framework for CKD. However, a more nationally-representative study is required to understand the etiologies of CKD among young adults especially women in Nigeria. The role of pregnancy-associated kidney disease, exposure to mercury containing cosmetic products, use of herbal remedy and CKD of unknown cause should be explored, and for an improved prevalence estimate of CKD in Nigeria.

## Data Availability

The dataset used and analyzed during the study is available from the Ilorin renal study group through the corresponding author on reasonable request.

## Abbreviations

aOR: Adjusted odds ratio
BMI: Body mass index
CG: Cochroft-Gault
CI: Confidence interval
CKD: Chronic kidney disease
CKD-EPI: Chronic kidney disease epidemiology collaboration
CVD: Cardiovascular disease
eGFR: Estimated glomerular filtration rate
ESRD: End-stage renal disease
GBD: Global burden of disease
HC: Hip circumference
IQR: Interquartile range
IRSG: Ilorin renal study group
LGA: Local government areas
MDRD: Modification of Diet in Renal Disease
NHANES: National health and nutrition examination survey
OR: Odds ratio
SD: Standard deviation
SPSS: Statistical package for social sciences
SSA: Sub-Saharan Africa
SBP: Systolic blood pressure
WC: Waist circumference
WHR: Waist-hip ratio
